# Synthesis, crystal structure and properties of bis­(iso­seleno­cyanato-κ*N*)tetra­kis­(4-meth­oxy­pyridine-κ*N*)cobalt(II)

**DOI:** 10.1107/S2056989023001391

**Published:** 2023-02-21

**Authors:** Christian Näther, Inke Jess

**Affiliations:** a Institut für Anorganische Chemie, Universität Kiel, Max-Eyth-Str. 2, 24118 Kiel, Germany; University of Kentucky, USA

**Keywords:** crystal structure, nickel seleno­cyanate, discrete complex, thermal properties

## Abstract

Reaction of CoCl_2_·6H_2_O with KNCSe and 4-meth­oxy­pyridine in water led to the formation of the title compound, [Co(NCSe)_2_(C_6_H_7_NO)_4_] or Co(NCSe)_2_(4-meth­oxy­pyridine)_2_, which was characterized by single-crystal X-ray diffraction. Its asymmetric unit consists of one crystallographically independent Co cation, two seleno­cyanate anions and four 4-meth­oxy­pyridine coligands in general positions. In the crystal structure, the cobalt cations are sixfold coordinated by two terminal N-bonded seleno­cyanate anions and four 4-meth­oxy­pyridine coligands within a slightly distorted octa­hedral coordination. Between the complexes, weak C—H⋯Se inter­actions are found. IR spectroscopic investigations revealed that the CN stretching vibration of the anionic ligands is observed at 2068 cm^−1^, which is in agreement with the presence of only terminally coordinated seleno­cyanate anions. PXRD measurements prove that a pure compound was obtained. Differential thermoanalysis coupled to thermogravimetry (DTA-TG) at different heating rates shows that the TG curves are poorly resolved. PXRD measurements of the residue obtained by a TG measurement prove that an amorphous compound was obtained.

## Abstract 2

In the crystal structure of the title compound, the cobalt cations are sixfold coordinated by two terminal N-bonded seleno­cyanate anions and four 4-meth­oxy­pyridine coligands within a slightly distorted octa­hedral coordination.

## 1. Chemical context

Coordination compounds based on transition-metal thio­cyanates show versatile structural behavior (Buckingham, 1994 ▸; Barnett *et al.*, 2002 ▸; Werner *et al.*, 2015 ▸) and promising magnetic properties, because this ligand is able to mediate reasonable magnetic exchange (Barasiński *et al.*, 2010 ▸; Palion-Gazda *et al.*, 2015 ▸; Mousavi *et al.*, 2020 ▸). In this context, compounds based on Co(NCS)_2_ are of special inter­est because they can show inter­esting magnetic behavior, such as, for example, slow relaxations of the magnetization, which is indicative of single-chain magnetism (Lescouëzec *et al.*, 2005 ▸; Sun *et al.*, 2010 ▸; Dhers *et al.*, 2015 ▸). For the synthesis of such compounds, the Co^II^ cations must be linked *via* the anionic ligands into mono-periodic or di-periodic networks. Compounds with di-periodicity are rare; the majority of compounds being mono-periodic, in which the Co^II^ cations are octa­hedrally coordinated and linked into chains by pairs of anionic ligands (Guang *et al.*, 2007 ▸; Shi *et al.*, 2007 ▸; Shurdha *et al.*, 2013 ▸; Prananto *et al.*, 2017 ▸). If the chains are linear, ferromagnetic ordering (Werner *et al.*, 2014 ▸) or single-chain magnet behavior (Mautner *et al.*, 2018 ▸) is observed and if they are corrugated or exhibit an alternating Co coordination, the magnetic exchange is weak or completely suppressed (Dockum *et al.*, 1983 ▸; Böhme *et al.*, 2020 ▸, 2022 ▸). All this is well investigated for Co(NCS)_2_ compounds but not much is known for compounds based on Co(NCSe)_2_, because only two compounds with μ-1,3-bridging seleno­cyanate anions are reported in the literature (Boeckmann *et al.*, 2011 ▸; Wöhlert *et al.*, 2012 ▸; Neumann *et al.*, 2019 ▸). First results indicate that they behave in a similar manner to their thio­cyanate analogs and that the exchange of thio- by seleno­cyanate leads to an increase in the magnetic intra­chain inter­actions (Neumann *et al.*, 2019 ▸). 
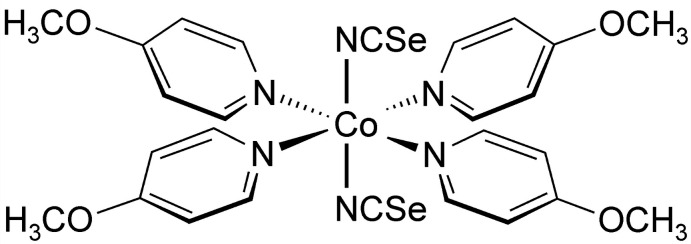


Unfortunately, the synthesis of compounds in which Co^II^ cations are linked by seleno­cyanate anions into chains in solution is always difficult to achieve or even impossible, because Co^II^ is not very chalcophilic and therefore, in most cases, compounds with terminally coordinated seleno­cyanate anions are obtained. To overcome this problem, we have developed an alternative approach for the synthesis of coordination networks based on thermal ligand removal of suitable precursor compounds that can be used for the synthesis of a wide range of materials including thio- and seleno­cyanates but also halide coordination compounds (Werner *et al.*, 2014 ▸; Boeckmann *et al.*, 2011 ▸; Näther & Jess, 2004 ▸). For thio­cyanate compounds, the precursors usually consist of discrete complexes of the general formula Co(NCS)_2_(*L*)_4_ (*L* = mono-coordinating coligand), in which the Co^II^ cations are octa­hedrally coordinated by two terminal N-bonded thio­cyanate anions and four coligands. Upon heating, most of compounds of this type lose half of the ligands in the first mass loss and the octa­hedral metal coordination is retained by the sulfur atoms that were not involved in the metal coordination in the precursor, which enforces the formation of compounds with bridging anionic ligands.

In the course of our systematic work we became inter­ested in the corresponding Co(NCSe)_2_ compounds with 4-meth­oxy­pyridine as coligand, because its thio­cyanate analog Co(NCS)_2_(4-meth­oxy­pyridine)_2_ crystallizes in the desired chain structure and is well investigated. This compound shows a metamagnetic transition and single-chain relaxations and this was investigated on powders but also using single crystals (Rams *et al.*, 2020 ▸; Foltyn *et al.*, 2022 ▸). The reaction of CoCl_2_·6H_2_O, KSeCN with 4-meth­oxy­pyridine in water, however, always led to the formation of a compound with the composition Co(NCSe)_2_(4-meth­oxy­pyridine)_4_ (see *Synthesis and crystallization*) for which the CN stretching vibration of the anionic ligand is observed at 2068 cm^−1^, indicative for the presence of only terminally bonded seleno­cyanate anions (Fig. S1). Even if CoCl_2_·6H_2_O and KSeCN were used in excess, no other crystalline phase was obtained. To identify this phase unambiguously, single crystals were grown and characterized by single-crystal X-ray diffraction.

## 2. Structural commentary

**Figure 1:**
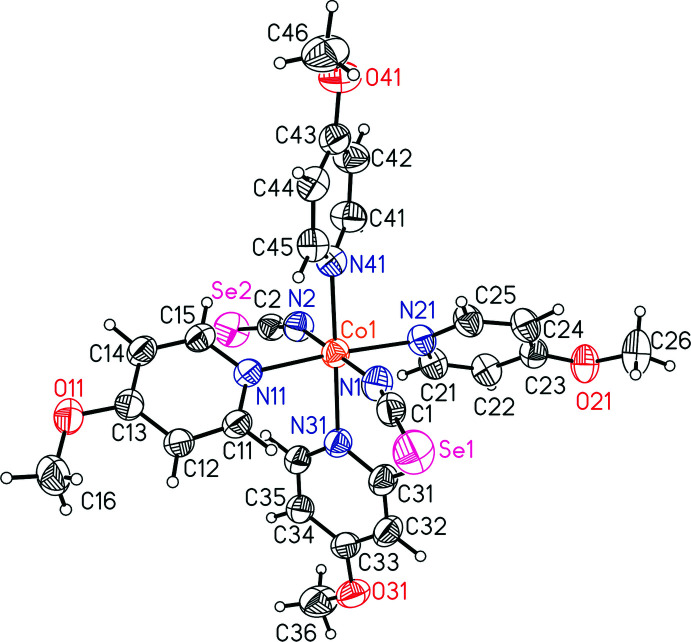
Crystal structure of the title compound with labeling and displacement ellipsoids drawn at the 50% probability level.

Single-crystal structure determination proved that the title compound, Co(NCSe)_2_(4-meth­oxy­pyridine)_4_, consists of discrete complexes in which the Co cations are sixfold coordinated to four 4-meth­oxy­pyridine coligands and two terminal seleno­cyanate anions that coordinate *via* the N atom of the anionic ligand to the metal center (Fig. 1 ▸). The asymmetric unit consists of one Co^II^ cation, two seleno­cyanate anions and four 4-meth­oxy­pyridine ligands in general positions. From the bond lengths and angles, it is obvious that the octa­hedra are slightly distorted (Table 1 ▸). This is also obvious from the angle variance of 1.77 and the quadratic elongation of 1.00 calculated using the method of Robinson (Robinson *et al.*, 1971 ▸).

The title compound is isotypic to *M*(NCS)_2_(4-meth­oxy­pyridine) (*M* = Co, Fe, Ni) already described in the literature (Mautner *et al.*, 2018 ▸; Jochim *et al.*, 2018 ▸). In this context, it is noted that Ni(NCS)_2_(4-meth­oxy­pyridine) crystallizes in two polymorphic modifications, of which the form (ortho­rhom­bic, space group *Pccn*) that is not isotypic to the title compound and *M*(NCS)_2_(4-meth­oxy­pyridine (*M* = Co, Fe) represents the thermodynamically stable phase at room temperature (Jochim *et al.*, 2018 ▸). However, from this experimental observation one cannot conclude that the title compound is metastable at room temperature and that a second form must exist. It is also noted that the thio­cyanate analogs with manganese and cadmium crystallize in a third form (monoclinic, space group *C*2/*c*) and that the Cd(NCS)_2_ compound also shows dimorphism and additionally crystallizes in a fourth form (tetra­gonal, space group *P*4_1_), which shows the pronounced structural variability for such simple complexes (Jochim *et al.*, 2019 ▸). Finally, it is noted that we have not found any evidence that the title compound crystallizes in a further crystalline form.

## 3. Supra­molecular features

**Figure 2:**
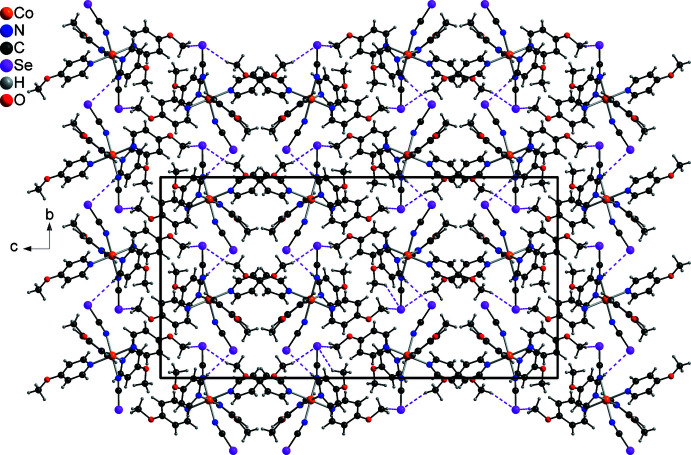
Crystal structure of the title compound viewed along the crystallographic *a-*axis direction. C—H⋯Se inter­actions are shown as pink dashed lines.

In the crystal structure, the discrete complexes are arranged in an irregular manner (Fig. 2 ▸) There are a number of inter­molecular C—H⋯O and C—H⋯Se contacts, but for most of them the C—H⋯*X* (*X* = O, Se) angle is far from linear and the H⋯*X* distances are too large for any significant inter­action (Table 2 ▸). Some of C—H⋯Se contacts exhibit angles larger than 150°, which might point to some inter­action (Fig. 2 ▸ and Table 2 ▸).

## 4. Thermal properties

**Figure 3:**
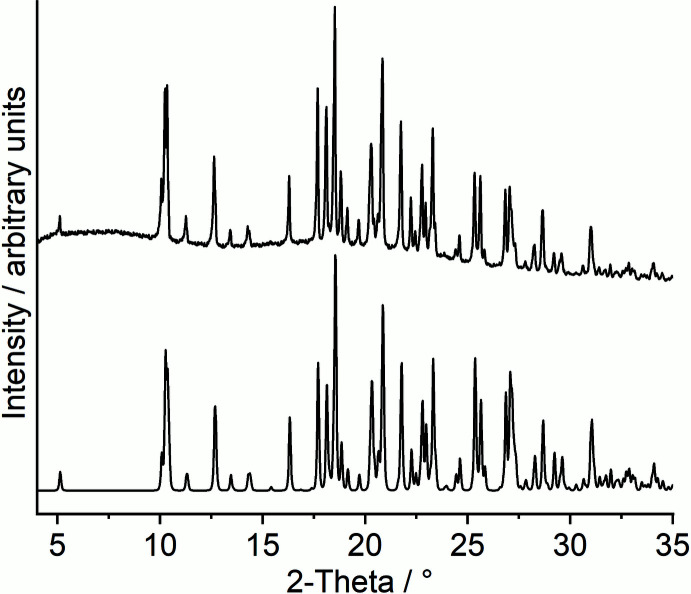
Experimental (top) and calculated PXRD pattern (bottom) of the title compound.

**Figure 4:**
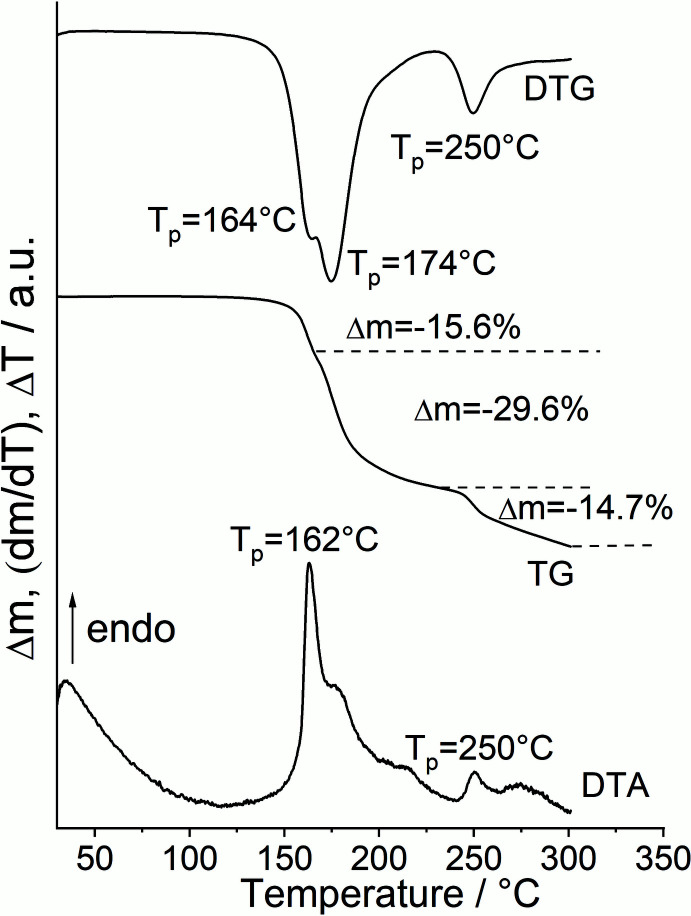
DTG, TG and DTA curves for the title compound measured at 8°C min^−1^ in a nitro­gen atmosphere.

Based on the single-crystal structural data, a powder pattern was calculated and compared with the experimental pattern, which shows that a pure crystalline phase was obtained (Fig. 3 ▸). To investigate if a crystalline ligand deficient phase with the composition Co(NCS)_2_(4-meth­oxy­pyridine)_4_ can be obtained, measurements using differential thermal analysis and thermogravimetry with 8°C min^−1^ were performed. Upon heating, the TG curve shows two poorly resolved mass losses at about 160 and 250°C that are accompanied with endothermic events in the DTA curve (Fig. 4 ▸). The DTG curve indicates that the first event consists of two different thermal events that cannot be successfully resolved. Nevertheless, the experimental mass loss was calculated for all three events, which shows that the first mass loss is in reasonable agreement with that calculated for the removal of one 4-meth­oxy­pyridine ligand (Δ*m* = −15.5%), whereas the second mass loss points to the removal of two additional 4-meth­oxy­pyridine ligands (Fig. 4 ▸). This would indicate that in the first step a compound with the composition Co(NCSe)_2_(4-meth­oxy­pyridine)_3_ is formed, which transforms into Co(NCSe)_2_(4-meth­oxy­pyridine) upon further heating. Compounds with such a ratio between the metal salt and neutral coligands are known for thio­cyanate coordination compounds, but are very rare for seleno­cyanates. One compound with the composition Ni(NCSe)_2_[*N*,*N*′-bis­(3-amino­prop­yl)methyl­amine]_2_ is found in which each Ni cation is octa­hedrally coordinated by three N atoms of one (3-amino­prop­yl)methyl­amine ligand plus two bridging and two terminal seleno­cyanate anions (Vicente *et al.*, 1993 ▸). Two of the Ni cations are linked by pairs of μ-1,3-bridging anionic ligands into dinculear units. At first glance, the Ni:coligand ratio seems to be different but one (3-amino­prop­yl)methyl­amine ligand replaces three monocoordinating ligands. For a ratio of 1:1 between *M*(NCS)_2_ and coligand, no examples can be found with seleno­cyanate anions but a few examples with thio­cyanate are reported in the literature, including Ni(NCS)_2_(4-amino­pyridine), in which Ni(NCS)_2_ double chains are observed (Neumann *et al.*, 2018 ▸).

To increase the resolution, measurements at different heating rates were performed, but the TG curves look similar and are still poorly resolved (Fig. S2). However, to investigate if different crystalline phases can be prepared, the residues obtained at different temperatures were isolated and investigated by PXRD, which proved that they are amorphous, and in Fig. S3 one of these patterns is shown as a representative. We also tried to anneal samples of the title compound at constant temperatures but always obtained amorphous inter­mediates. Therefore, no more efforts were made.

## 5. Database survey

In the CCDC database, no seleno­cyanate compounds with 4-meth­oxy­pyridine are reported (CSD version 5.42, last update November 2021; Groom *et al.*, 2016 ▸), but some compounds with thio­cyanate as the anionic ligand are found. They include compounds with the composition *M*(NCS)_2_(4-meth­oxy­pyridine)_4_ with *M* = Mn (Refcode COBVEX; Jochim *et al.*, 2019 ▸), Fe (Refcode FISCIW; Jochim *et al.*, 2018 ▸), Co (Refcode KIJPUR; Mautner *et al.*, 2018 ▸), Ni (Refcodes FISCAO and FISCES; Jochim *et al.*, 2019 ▸), Cd (Refcode COBTUL and COBTUL01; Jochim *et al.*, 2019 ▸) and Ru (Refcode NAGPOD; Cadranel *et al.*, 2016 ▸), which form discrete complexes with octa­hedral coordination. All of these compounds crystallize in four different structure types. There are additional discrete octa­hedral complexes with the composition Cd(NCS)_2_(4-meth­oxy­pyridine)_2_·4-meth­oxy­pyri­dine (Refcode COBVAT; Jochim *et al.*, 2019 ▸) and Ni(NCS)_2_(4-meth­oxy­pyridine)_2_·aceto­nitrile (Refcode FISCOC; Jochim *et al.*, 2018 ▸) that form solvates and one aceto­nitrile complex with the composition Ni(NCS)_2_(4-meth­oxy­pyridine)_2_(CH_3_CN)_2_ (Refcode FISCES; Jochim *et al.*, 2018 ▸).

With thio­cyanate, compounds are reported with the composition *M*(NCS)_2_(4-meth­oxy­pyridine)_2_ with *M* = Cu (Refcode ABOXAT; Handy *et al.*, 2017 ▸), Co (KIJQAY, KIJPOL and KIJPOL01; Mautner *et al.*, 2018 ▸ and Rams *et al.*, 2020 ▸), Ni (FISBUH; Jochim *et al.*, 2018 ▸), Cd (COBTUL and COBVIB; Jochim *et al.*, 2019 ▸). The Cu compound forms discrete complexes with a square-planar coordination, while the Co compounds consist of isomers forming discrete tetra­hedral complexes as well as a chain compound with an octa­hedral coordination, which is also the case for the Co and Cd compounds.

There are also discrete complexes with seleno­cyanate anions and pyridine derivatives as coligands reported in the literature that are comparable to the title compound. These include, for example, Fe(NCSe)_2_[4-2(phenyl­vin­yl)pyridine-N]_4_ (Refcodes XUKNUN, XUKNUN01, XUKPEZ and XUKPEZ01; Boillot *et al.*, 2009 ▸) and Co(NCSe)_2_ [Refcodes ITISOU (Boeckmann & Näther, 2011 ▸) and TIXDOW, TIXDOW01 and TIXFAK (Neumann *et al.*, 2019 ▸)].

## 6. Synthesis and crystallization

CoCl_2_·6H_2_O and KSeCN were purchased from Aldrich and 4-meth­oxy­pyridine was purchased from Alfa Aesar.

**Synthesis:**

Larger amounts of a microcrystalline powder were obtained by the reaction of 0.15 mmol (35.7 mg) of CoCl_2_·6H_2_O with 0.30 mmol (43.3 mg) of KSeCN and 0.60 mmol (60.8 µL) of 4-meth­oxy­pyridine in 1 ml of demineralized water. The mixture was stirred for 2 d at room temperature, the light-pink-colored precipitate was filtered off and washed with a very small amount of water. Single crystals were obtained by slow evaporation of the solvent from the filtrate. It is noted that the same compound is obtained if CoCl_2_·6H_2_O and 4-meth­oxy­pyridine are used in a 1:1 ratio.

**Experimental details:**

The XRPD measurements were performed with a Stoe Transmission Powder Diffraction System (STADI P) equipped with a MYTHEN 1K detector and a Johansson-type Ge(111) monochromator using Cu *K*α_1_ radiation (λ = 1.540598 Å). The IR spectra were measured using an ATI Mattson Genesis Series FTIR Spectrometer, control software: *WINFIRST*, from ATI Mattson. Thermogravimetry and differential thermoanalysis (TG–DTA) measurements were performed in a dynamic nitro­gen atmosphere in Al_2_O_3_ crucibles using a STA-PT 1000 thermobalance from Linseis. The instrument was calibrated using standard reference materials.

## 7. Refinement

Crystal data, data collection and structure refinement details are summarized in Table 3 ▸. Hydrogen atoms were positioned with idealized geometry (C—H = 0.95–0.98 Å, methyl H atoms allowed to rotate but not to tip) and were refined with *U*_iso_(H) = 1.2*U*_eq_(C) (1.5 for methyl H atoms) using a riding model.

## Supplementary Material

Crystal structure: contains datablock(s) I. DOI: 10.1107/S2056989023001391/pk2678sup1.cif

Structure factors: contains datablock(s) I. DOI: 10.1107/S2056989023001391/pk2678Isup2.hkl

Fig. S1. IR spectrum of the title compound. Given is the value of the CN stretching vibration of the selenocyanate anions. DOI: 10.1107/S2056989023001391/pk2678sup3.jpg

Fig. S2. Thermogravimetric curves of the title compound at 1, 4 and 8 C/min. DOI: 10.1107/S2056989023001391/pk2678sup4.jpg

Fig. S3. Experimental PXRD pattern of the residue obtained from a TG-DTA measurement of the title compound with 8 C/min at about 240 C. DOI: 10.1107/S2056989023001391/pk2678sup5.jpg

CCDC reference: 2242297

Additional supporting information:  crystallographic information; 3D view; checkCIF report

## Figures and Tables

**Table 1:** Selected geometric parameters (Å, °)

Co1—N2	2.092 (2)	Co1—N21	2.170 (2)
Co1—N1	2.108 (2)	Co1—N41	2.174 (2)
Co1—N11	2.139 (2)	Co1—N31	2.203 (2)
			
N2—Co1—N1	178.93 (10)	N11—Co1—N41	91.26 (8)
N2—Co1—N11	90.65 (9)	N21—Co1—N41	92.61 (9)
N1—Co1—N11	90.00 (9)	N2—Co1—N31	88.71 (9)
N2—Co1—N21	90.08 (9)	N1—Co1—N31	90.47 (9)
N1—Co1—N21	89.20 (9)	N11—Co1—N31	88.32 (8)
N11—Co1—N21	176.06 (9)	N21—Co1—N31	87.83 (9)
N2—Co1—N41	90.07 (9)	N41—Co1—N31	178.70 (9)
N1—Co1—N41	90.76 (9)		

**Table 2:** Hydrogen-bond geometry (Å, °)

*D*—H⋯*A*	*D*—H	H⋯*A*	*D*⋯*A*	*D*—H⋯*A*
C11—H11⋯O11^i^	0.95	2.49	3.165 (3)	128
C15—H15⋯O31^ii^	0.95	2.52	3.297 (3)	139
C16—H16*B*⋯Se1^iii^	0.98	3.15	4.096 (3)	163
C22—H22⋯Se1^iv^	0.95	3.12	3.817 (3)	132
C26—H26*A*⋯Se1^v^	0.98	3.06	4.029 (5)	171
C36—H36*B*⋯Se2^vi^	0.98	3.13	3.952 (4)	142
C41—H41⋯O21^vii^	0.95	2.43	3.248 (4)	144
C45—H45⋯Se2^viii^	0.95	3.08	3.932 (3)	151
C46—H46*A*⋯Se1^ii^	0.98	3.15	3.885 (4)	133

Symmetry codes: (i) 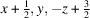 ; (ii) 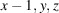 ; (iii) 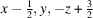 ; (iv) 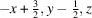 ; (v) 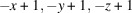 ; (vi) 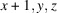 ; (vii) 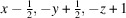 ; (viii) 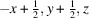 .

**Table 3:** Experimental details

Crystal data	
Chemical formula	[Co(NCSe)_2_(C_6_H_7_NO)_4_]
*M*_r_	705.39
Crystal system, space group	Orthorhombic, *P* *b* *c* *a*
Temperature (K)	200
*a*, *b*, *c* (Å)	10.0531 (2), 17.3479 (4), 34.3141 (5)
*V* (Å^3^)	5984.4 (2)
*Z*	8
Radiation type	Mo *K*α
μ (mm^−1^)	3.05
Crystal size (mm)	0.23 × 0.19 × 0.17

Data collection	
Diffractometer	Stoe *IPDS2*
Absorption correction	Numerical (*X-RED* and *X-SHAPE*; Stoe, 2008 ▸)
*T*_min_, *T*_max_	0.457, 0.738
No. of measured, independent and observed [*I* > 2σ(*I*)] reflections	56525, 5862, 5130
*R*_int_	0.035
(sin θ/λ)_max_ (Å^−1^)	0.617

Refinement	
*R*[*F* ^2^ > 2σ(*F* ^2^)], *wR*(*F* ^2^), *S*	0.038, 0.087, 1.07
No. of reflections	5862
No. of parameters	356
H-atom treatment	H-atom parameters constrained
Δρ_max_, Δρ_min_ (e Å^−3^)	0.34, −0.48

Computer programs: *X-AREA* (Stoe, 2008 ▸), *SHELXT2014/5* (Sheldrick, 2015*a* ▸), *SHELXL2016/6* (Sheldrick, 2015*b* ▸), *DIAMOND* (Brandenburg & Putz, 1999 ▸) and *publCIF* (Westrip, 2010 ▸).

## supplementary crystallographic information

### Crystal data

**Table:**

[Co(NCSe)_2_(C_6_H_7_NO)_4_]	*D*_x_ = 1.566 Mg m^−^^3^
*M**_r_* = 705.39	Mo *K*α radiation, λ = 0.71073 Å
Orthorhombic, *P**b**c**a*	Cell parameters from 56525 reflections
*a* = 10.0531 (2) Å	θ = 1.2–26.0°
*b* = 17.3479 (4) Å	µ = 3.05 mm^−^^1^
*c* = 34.3141 (5) Å	*T* = 200 K
*V* = 5984.4 (2) Å^3^	Block, light pink
*Z* = 8	0.23 × 0.19 × 0.17 mm
*F*(000) = 2824	

### Data collection

**Table:**

Stoe IPDS-2 diffractometer	5130 reflections with *I* > 2σ(*I*)
ω scans	*R*_int_ = 0.035
Absorption correction: numerical (X-Red and X-Shape; Stoe, 2008)	θ_max_ = 26.0°, θ_min_ = 1.2°
*T*_min_ = 0.457, *T*_max_ = 0.738	*h* = −12→9
56525 measured reflections	*k* = −21→21
5862 independent reflections	*l* = −42→42

### Refinement

**Table:**

Refinement on *F*^2^	0 restraints
Least-squares matrix: full	Hydrogen site location: inferred from neighbouring sites
*R*[*F*^2^ > 2σ(*F*^2^)] = 0.038	H-atom parameters constrained
*wR*(*F*^2^) = 0.087	*w* = 1/[σ^2^(*F*_o_^2^) + (0.0382*P*)^2^ + 4.2748*P*] where *P* = (*F*_o_^2^ + 2*F*_c_^2^)/3
*S* = 1.07	(Δ/σ)_max_ = 0.003
5862 reflections	Δρ_max_ = 0.34 e Å^−^^3^
356 parameters	Δρ_min_ = −0.47 e Å^−^^3^

### Special details

**Table:**

Geometry. All esds (except the esd in the dihedral angle between two l.s. planes) are estimated using the full covariance matrix. The cell esds are taken into account individually in the estimation of esds in distances, angles and torsion angles; correlations between esds in cell parameters are only used when they are defined by crystal symmetry. An approximate (isotropic) treatment of cell esds is used for estimating esds involving l.s. planes.

### Fractional atomic coordinates and isotropic or equivalent isotropic displacement parameters (Å^2^)

**Table:**

	*x*	*y*	*z*	*U*_iso_*/*U*_eq_	
Co1	0.42296 (4)	0.38984 (2)	0.62207 (2)	0.04280 (10)	
N1	0.4898 (3)	0.50081 (14)	0.60651 (7)	0.0509 (5)	
C1	0.5320 (3)	0.56297 (17)	0.60594 (8)	0.0479 (6)	
Se1	0.59634 (4)	0.65844 (2)	0.60535 (2)	0.07361 (13)	
N2	0.3601 (2)	0.27886 (13)	0.63726 (7)	0.0504 (5)	
C2	0.3463 (3)	0.22263 (16)	0.65509 (8)	0.0453 (6)	
Se2	0.32769 (4)	0.13723 (2)	0.68306 (2)	0.06769 (12)	
N11	0.3567 (2)	0.43341 (13)	0.67705 (6)	0.0426 (5)	
C11	0.4297 (3)	0.48409 (16)	0.69698 (8)	0.0449 (6)	
H11	0.508929	0.502719	0.685193	0.054*	
C12	0.3978 (3)	0.51110 (16)	0.73342 (7)	0.0435 (6)	
H12	0.454554	0.546307	0.746578	0.052*	
C13	0.2809 (3)	0.48581 (15)	0.75052 (8)	0.0443 (6)	
C14	0.2031 (3)	0.43319 (16)	0.72997 (8)	0.0482 (6)	
H14	0.122509	0.414453	0.740865	0.058*	
C15	0.2436 (3)	0.40891 (15)	0.69422 (8)	0.0449 (6)	
H15	0.189538	0.372893	0.680651	0.054*	
O11	0.2361 (2)	0.50833 (14)	0.78569 (6)	0.0613 (5)	
C16	0.3177 (4)	0.5600 (2)	0.80767 (10)	0.0743 (10)	
H16A	0.402605	0.534966	0.813740	0.111*	
H16B	0.272280	0.573788	0.831961	0.111*	
H16C	0.334262	0.606763	0.792371	0.111*	
N21	0.5040 (2)	0.34562 (13)	0.56789 (6)	0.0469 (5)	
C21	0.5856 (3)	0.28430 (16)	0.56790 (8)	0.0496 (6)	
H21	0.587114	0.252310	0.590368	0.059*	
C22	0.6666 (3)	0.26539 (16)	0.53735 (8)	0.0512 (7)	
H22	0.722378	0.221281	0.538708	0.061*	
C23	0.6661 (3)	0.31148 (15)	0.50441 (8)	0.0475 (6)	
C24	0.5771 (3)	0.37243 (16)	0.50266 (8)	0.0523 (7)	
H24	0.569119	0.403015	0.479818	0.063*	
C25	0.5007 (3)	0.38721 (17)	0.53511 (8)	0.0510 (7)	
H25	0.441604	0.429897	0.534145	0.061*	
O21	0.7538 (2)	0.29286 (12)	0.47615 (6)	0.0593 (5)	
C26	0.7672 (4)	0.3449 (2)	0.44407 (10)	0.0761 (11)	
H26A	0.683599	0.346536	0.429403	0.114*	
H26B	0.839033	0.327220	0.426951	0.114*	
H26C	0.788117	0.396546	0.453862	0.114*	
N31	0.6165 (2)	0.36451 (13)	0.64936 (6)	0.0441 (5)	
C31	0.7328 (3)	0.38797 (17)	0.63423 (8)	0.0513 (7)	
H31	0.730644	0.422024	0.612555	0.062*	
C32	0.8543 (3)	0.36555 (18)	0.64827 (9)	0.0552 (7)	
H32	0.933751	0.384252	0.636632	0.066*	
C33	0.8602 (3)	0.31539 (17)	0.67957 (8)	0.0468 (6)	
C34	0.7418 (3)	0.29132 (16)	0.69587 (8)	0.0478 (6)	
H34	0.741410	0.257422	0.717608	0.057*	
C35	0.6246 (3)	0.31725 (17)	0.68008 (7)	0.0463 (6)	
H35	0.543881	0.300535	0.691747	0.056*	
O31	0.9822 (2)	0.29425 (13)	0.69180 (6)	0.0613 (5)	
C36	0.9907 (4)	0.2312 (2)	0.71856 (11)	0.0772 (10)	
H36A	0.949864	0.245955	0.743396	0.116*	
H36B	1.084362	0.217962	0.722851	0.116*	
H36C	0.943834	0.186517	0.707773	0.116*	
N41	0.2300 (2)	0.41270 (13)	0.59591 (6)	0.0471 (5)	
C41	0.1720 (3)	0.36068 (17)	0.57240 (9)	0.0562 (7)	
H41	0.214155	0.312096	0.569187	0.067*	
C42	0.0557 (3)	0.37370 (19)	0.55288 (9)	0.0592 (8)	
H42	0.019107	0.335190	0.536376	0.071*	
C43	−0.0080 (3)	0.44397 (18)	0.55750 (8)	0.0512 (6)	
C44	0.0467 (3)	0.49680 (16)	0.58295 (8)	0.0505 (7)	
H44	0.003580	0.544520	0.587894	0.061*	
C45	0.1652 (3)	0.47896 (15)	0.60105 (8)	0.0476 (6)	
H45	0.202896	0.516039	0.618178	0.057*	
O41	−0.1208 (2)	0.45430 (15)	0.53667 (7)	0.0696 (6)	
C46	−0.1853 (4)	0.5276 (2)	0.53937 (13)	0.0798 (11)	
H46A	−0.212437	0.536910	0.566395	0.120*	
H46B	−0.263923	0.528030	0.522492	0.120*	
H46C	−0.123661	0.568224	0.531068	0.120*	

### Atomic displacement parameters (Å^2^)

**Table:**

	*U*^11^	*U*^22^	*U*^33^	*U*^12^	*U*^13^	*U*^23^
Co1	0.04374 (19)	0.04212 (19)	0.04252 (19)	−0.00171 (15)	0.00192 (15)	0.00372 (14)
N1	0.0553 (14)	0.0453 (13)	0.0522 (13)	−0.0025 (11)	0.0056 (11)	0.0050 (10)
C1	0.0508 (16)	0.0524 (16)	0.0405 (13)	0.0026 (13)	0.0043 (12)	0.0047 (11)
Se1	0.0947 (3)	0.05033 (18)	0.0758 (2)	−0.02052 (18)	0.00388 (19)	0.00265 (15)
N2	0.0518 (14)	0.0460 (13)	0.0532 (13)	−0.0049 (11)	−0.0007 (11)	0.0044 (11)
C2	0.0431 (14)	0.0463 (15)	0.0465 (14)	−0.0031 (12)	−0.0012 (11)	−0.0067 (12)
Se2	0.0820 (3)	0.04693 (18)	0.0741 (2)	−0.00859 (16)	−0.00418 (18)	0.01474 (15)
N11	0.0373 (11)	0.0470 (12)	0.0435 (11)	−0.0019 (9)	0.0011 (9)	0.0020 (9)
C11	0.0371 (13)	0.0513 (15)	0.0463 (14)	−0.0056 (11)	0.0006 (11)	0.0047 (11)
C12	0.0393 (14)	0.0477 (14)	0.0436 (13)	−0.0030 (11)	−0.0023 (11)	0.0031 (11)
C13	0.0431 (14)	0.0473 (14)	0.0426 (13)	0.0031 (12)	0.0030 (11)	0.0028 (11)
C14	0.0386 (14)	0.0539 (16)	0.0523 (15)	−0.0053 (12)	0.0053 (12)	0.0034 (12)
C15	0.0377 (13)	0.0459 (14)	0.0511 (14)	−0.0042 (11)	0.0014 (11)	0.0028 (11)
O11	0.0571 (12)	0.0760 (14)	0.0509 (11)	−0.0092 (11)	0.0127 (10)	−0.0123 (10)
C16	0.082 (2)	0.082 (2)	0.0585 (19)	−0.018 (2)	0.0122 (17)	−0.0227 (17)
N21	0.0525 (14)	0.0458 (12)	0.0424 (11)	0.0030 (10)	0.0003 (10)	0.0025 (9)
C21	0.0608 (17)	0.0426 (14)	0.0452 (14)	0.0043 (13)	0.0002 (12)	0.0034 (11)
C22	0.0617 (18)	0.0419 (14)	0.0499 (15)	0.0077 (13)	−0.0003 (13)	−0.0001 (12)
C23	0.0534 (16)	0.0433 (14)	0.0457 (14)	−0.0035 (12)	0.0041 (12)	−0.0052 (11)
C24	0.0649 (18)	0.0483 (15)	0.0436 (14)	0.0069 (13)	−0.0003 (13)	0.0044 (12)
C25	0.0568 (17)	0.0528 (16)	0.0434 (14)	0.0098 (13)	−0.0011 (13)	0.0033 (12)
O21	0.0743 (14)	0.0504 (11)	0.0532 (11)	0.0053 (10)	0.0172 (10)	−0.0017 (9)
C26	0.101 (3)	0.0597 (19)	0.067 (2)	0.0058 (19)	0.035 (2)	0.0068 (16)
N31	0.0420 (12)	0.0459 (12)	0.0445 (11)	−0.0011 (10)	0.0022 (9)	0.0036 (9)
C31	0.0481 (16)	0.0540 (16)	0.0520 (15)	−0.0062 (13)	0.0044 (12)	0.0114 (13)
C32	0.0437 (15)	0.0617 (18)	0.0601 (17)	−0.0075 (14)	0.0074 (13)	0.0104 (14)
C33	0.0404 (14)	0.0499 (15)	0.0501 (14)	−0.0031 (12)	−0.0030 (12)	−0.0017 (12)
C34	0.0472 (15)	0.0512 (15)	0.0450 (14)	−0.0061 (12)	−0.0002 (12)	0.0063 (12)
C35	0.0415 (14)	0.0542 (15)	0.0432 (13)	−0.0051 (12)	0.0035 (11)	0.0063 (12)
O31	0.0428 (11)	0.0712 (14)	0.0700 (13)	−0.0044 (10)	−0.0064 (10)	0.0116 (11)
C36	0.058 (2)	0.090 (3)	0.084 (2)	0.0044 (19)	−0.0175 (18)	0.027 (2)
N41	0.0496 (13)	0.0450 (12)	0.0466 (12)	0.0013 (10)	−0.0008 (10)	0.0004 (9)
C41	0.0558 (18)	0.0505 (16)	0.0623 (17)	0.0076 (14)	−0.0092 (14)	−0.0121 (13)
C42	0.0569 (18)	0.0586 (18)	0.0620 (18)	0.0030 (14)	−0.0083 (14)	−0.0128 (14)
C43	0.0437 (15)	0.0586 (17)	0.0513 (15)	0.0013 (13)	0.0005 (12)	0.0066 (13)
C44	0.0502 (16)	0.0450 (14)	0.0563 (16)	0.0032 (12)	0.0064 (13)	0.0053 (12)
C45	0.0524 (16)	0.0408 (13)	0.0494 (15)	−0.0002 (12)	0.0019 (12)	0.0016 (11)
O41	0.0531 (13)	0.0749 (15)	0.0807 (15)	0.0082 (12)	−0.0151 (11)	0.0034 (12)
C46	0.061 (2)	0.076 (2)	0.102 (3)	0.0171 (18)	−0.013 (2)	0.017 (2)

### Geometric parameters (Å, º)

**Table:**

Co1—N2	2.092 (2)	C25—H25	0.9500
Co1—N1	2.108 (2)	O21—C26	1.430 (4)
Co1—N11	2.139 (2)	C26—H26A	0.9800
Co1—N21	2.170 (2)	C26—H26B	0.9800
Co1—N41	2.174 (2)	C26—H26C	0.9800
Co1—N31	2.203 (2)	N31—C35	1.338 (3)
N1—C1	1.159 (4)	N31—C31	1.342 (4)
C1—Se1	1.778 (3)	C31—C32	1.370 (4)
N2—C2	1.160 (3)	C31—H31	0.9500
C2—Se2	1.775 (3)	C32—C33	1.384 (4)
N11—C11	1.334 (3)	C32—H32	0.9500
N11—C15	1.349 (3)	C33—O31	1.347 (3)
C11—C12	1.373 (4)	C33—C34	1.379 (4)
C11—H11	0.9500	C34—C35	1.373 (4)
C12—C13	1.384 (4)	C34—H34	0.9500
C12—H12	0.9500	C35—H35	0.9500
C13—O11	1.346 (3)	O31—C36	1.431 (4)
C13—C14	1.393 (4)	C36—H36A	0.9800
C14—C15	1.359 (4)	C36—H36B	0.9800
C14—H14	0.9500	C36—H36C	0.9800
C15—H15	0.9500	N41—C45	1.333 (3)
O11—C16	1.431 (4)	N41—C41	1.344 (4)
C16—H16A	0.9800	C41—C42	1.366 (4)
C16—H16B	0.9800	C41—H41	0.9500
C16—H16C	0.9800	C42—C43	1.386 (4)
N21—C25	1.337 (3)	C42—H42	0.9500
N21—C21	1.343 (3)	C43—O41	1.353 (3)
C21—C22	1.367 (4)	C43—C44	1.380 (4)
C21—H21	0.9500	C44—C45	1.379 (4)
C22—C23	1.384 (4)	C44—H44	0.9500
C22—H22	0.9500	C45—H45	0.9500
C23—O21	1.350 (3)	O41—C46	1.431 (4)
C23—C24	1.386 (4)	C46—H46A	0.9800
C24—C25	1.377 (4)	C46—H46B	0.9800
C24—H24	0.9500	C46—H46C	0.9800
			
N2—Co1—N1	178.93 (10)	N21—C25—H25	117.8
N2—Co1—N11	90.65 (9)	C24—C25—H25	117.8
N1—Co1—N11	90.00 (9)	C23—O21—C26	117.6 (2)
N2—Co1—N21	90.08 (9)	O21—C26—H26A	109.5
N1—Co1—N21	89.20 (9)	O21—C26—H26B	109.5
N11—Co1—N21	176.06 (9)	H26A—C26—H26B	109.5
N2—Co1—N41	90.07 (9)	O21—C26—H26C	109.5
N1—Co1—N41	90.76 (9)	H26A—C26—H26C	109.5
N11—Co1—N41	91.26 (8)	H26B—C26—H26C	109.5
N21—Co1—N41	92.61 (9)	C35—N31—C31	115.9 (2)
N2—Co1—N31	88.71 (9)	C35—N31—Co1	120.72 (18)
N1—Co1—N31	90.47 (9)	C31—N31—Co1	122.96 (18)
N11—Co1—N31	88.32 (8)	N31—C31—C32	123.7 (3)
N21—Co1—N31	87.83 (9)	N31—C31—H31	118.2
N41—Co1—N31	178.70 (9)	C32—C31—H31	118.2
C1—N1—Co1	166.1 (2)	C31—C32—C33	119.3 (3)
N1—C1—Se1	179.7 (3)	C31—C32—H32	120.3
C2—N2—Co1	160.3 (2)	C33—C32—H32	120.3
N2—C2—Se2	178.8 (3)	O31—C33—C34	125.2 (3)
C11—N11—C15	116.6 (2)	O31—C33—C32	116.9 (3)
C11—N11—Co1	120.90 (17)	C34—C33—C32	117.9 (3)
C15—N11—Co1	122.44 (18)	C35—C34—C33	118.8 (2)
N11—C11—C12	124.3 (2)	C35—C34—H34	120.6
N11—C11—H11	117.9	C33—C34—H34	120.6
C12—C11—H11	117.9	N31—C35—C34	124.3 (3)
C11—C12—C13	118.4 (2)	N31—C35—H35	117.8
C11—C12—H12	120.8	C34—C35—H35	117.8
C13—C12—H12	120.8	C33—O31—C36	117.6 (2)
O11—C13—C12	124.9 (3)	O31—C36—H36A	109.5
O11—C13—C14	117.1 (2)	O31—C36—H36B	109.5
C12—C13—C14	118.0 (2)	H36A—C36—H36B	109.5
C15—C14—C13	119.4 (2)	O31—C36—H36C	109.5
C15—C14—H14	120.3	H36A—C36—H36C	109.5
C13—C14—H14	120.3	H36B—C36—H36C	109.5
N11—C15—C14	123.3 (3)	C45—N41—C41	116.5 (2)
N11—C15—H15	118.4	C45—N41—Co1	122.59 (19)
C14—C15—H15	118.4	C41—N41—Co1	120.87 (19)
C13—O11—C16	117.5 (2)	N41—C41—C42	123.7 (3)
O11—C16—H16A	109.5	N41—C41—H41	118.1
O11—C16—H16B	109.5	C42—C41—H41	118.1
H16A—C16—H16B	109.5	C41—C42—C43	119.0 (3)
O11—C16—H16C	109.5	C41—C42—H42	120.5
H16A—C16—H16C	109.5	C43—C42—H42	120.5
H16B—C16—H16C	109.5	O41—C43—C44	125.5 (3)
C25—N21—C21	116.3 (2)	O41—C43—C42	116.3 (3)
C25—N21—Co1	121.38 (19)	C44—C43—C42	118.2 (3)
C21—N21—Co1	120.60 (18)	C45—C44—C43	118.7 (3)
N21—C21—C22	123.6 (3)	C45—C44—H44	120.6
N21—C21—H21	118.2	C43—C44—H44	120.6
C22—C21—H21	118.2	N41—C45—C44	123.8 (3)
C21—C22—C23	119.0 (3)	N41—C45—H45	118.1
C21—C22—H22	120.5	C44—C45—H45	118.1
C23—C22—H22	120.5	C43—O41—C46	117.6 (3)
O21—C23—C22	116.5 (3)	O41—C46—H46A	109.5
O21—C23—C24	125.0 (3)	O41—C46—H46B	109.5
C22—C23—C24	118.6 (3)	H46A—C46—H46B	109.5
C25—C24—C23	117.8 (3)	O41—C46—H46C	109.5
C25—C24—H24	121.1	H46A—C46—H46C	109.5
C23—C24—H24	121.1	H46B—C46—H46C	109.5
N21—C25—C24	124.5 (3)		

### Hydrogen-bond geometry (Å, º)

**Table:**

*D*—H···*A*	*D*—H	H···*A*	*D*···*A*	*D*—H···*A*
C11—H11···O11^i^	0.95	2.49	3.165 (3)	128
C15—H15···O31^ii^	0.95	2.52	3.297 (3)	139
C16—H16*B*···Se1^iii^	0.98	3.15	4.096 (3)	163
C22—H22···Se1^iv^	0.95	3.12	3.817 (3)	132
C26—H26*A*···Se1^v^	0.98	3.06	4.029 (5)	171
C36—H36*B*···Se2^vi^	0.98	3.13	3.952 (4)	142
C41—H41···O21^vii^	0.95	2.43	3.248 (4)	144
C45—H45···Se2^viii^	0.95	3.08	3.932 (3)	151
C46—H46*A*···Se1^ii^	0.98	3.15	3.885 (4)	133

Symmetry codes: (i) *x*+1/2, *y*, −*z*+3/2; (ii) *x*−1, *y*, *z*; (iii) *x*−1/2, *y*, −*z*+3/2; (iv) −*x*+3/2, *y*−1/2, *z*; (v) −*x*+1, −*y*+1, −*z*+1; (vi) *x*+1, *y*, *z*; (vii) *x*−1/2, −*y*+1/2, −*z*+1; (viii) −*x*+1/2, *y*+1/2, *z*.
