# Supplementary figures and images for: Synthesis, crystal structure and properties of bis­(iso­seleno­cyanato-κN)tetra­kis­(4-meth­oxy­pyridine-κN)cobalt(II)
Source: Acta Crystallogr E Crystallogr Commun. 2023 Feb 21;79(Pt 3):216–20. doi: 10.1107/S2056989023001391 (PMC9993910; doi:10.1107/S2056989023001391)

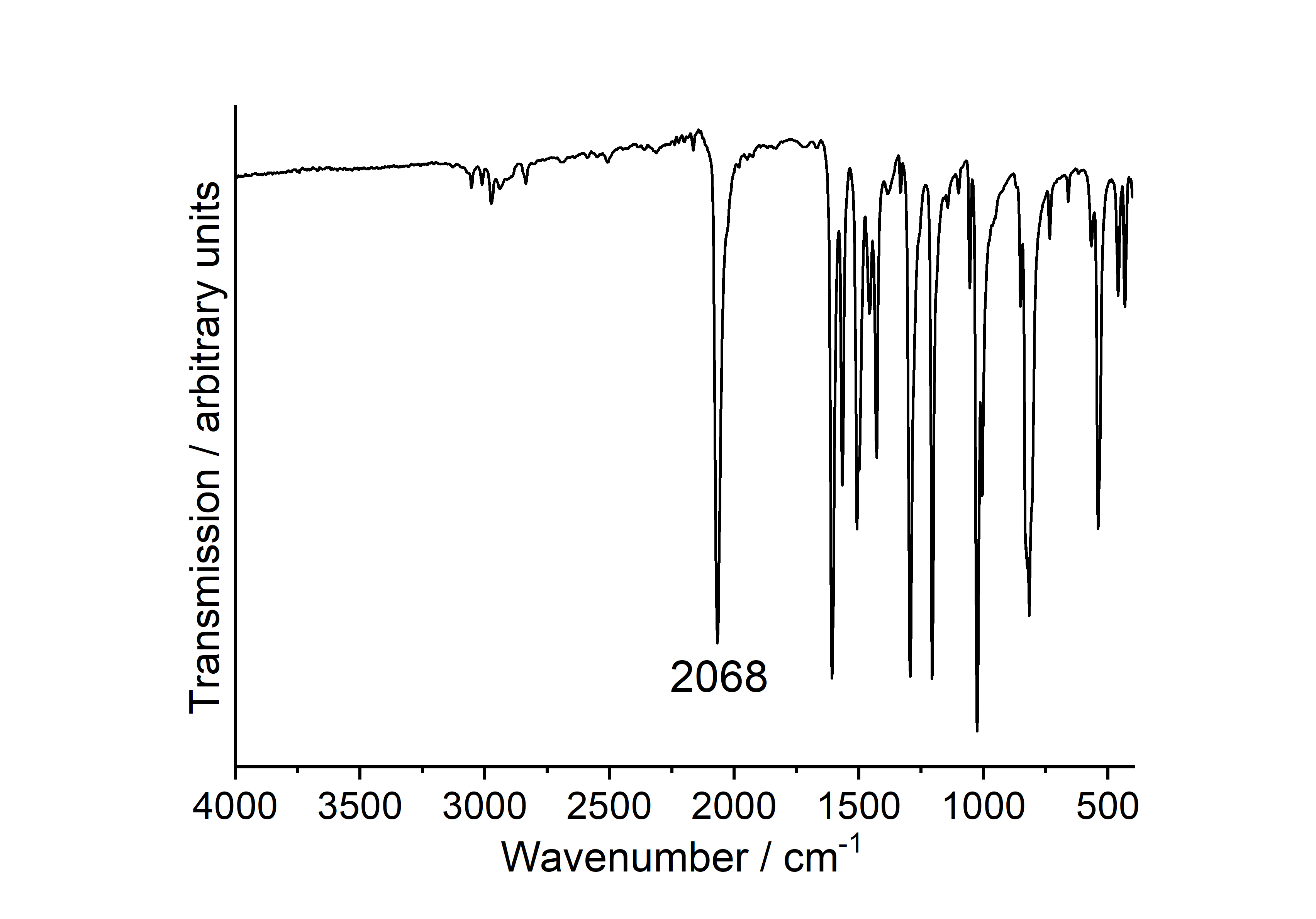

Supplement: Supplementary file 3 [file e-79-00216-sup3.jpg]

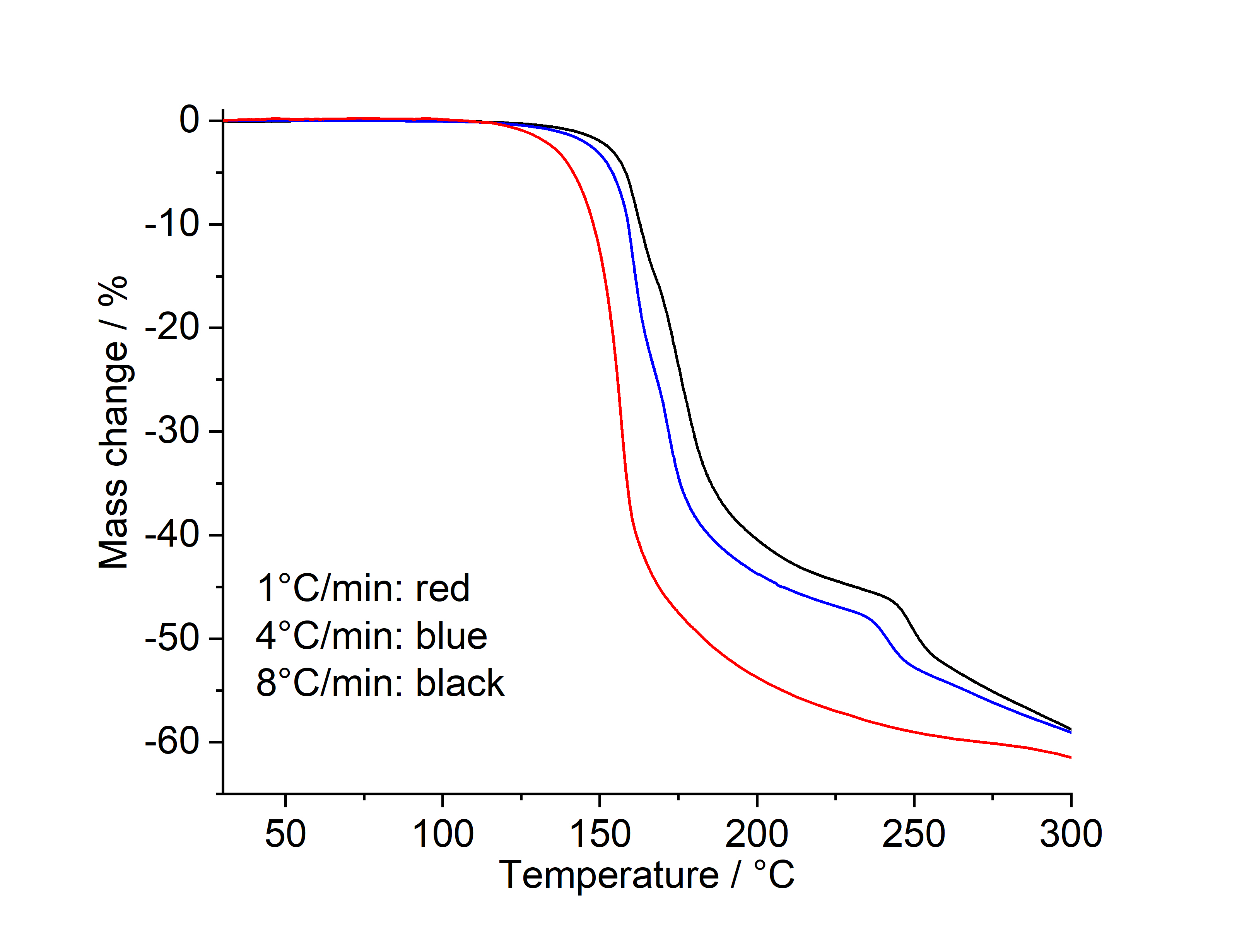

Supplement: Supplementary file 4 [file e-79-00216-sup4.jpg]

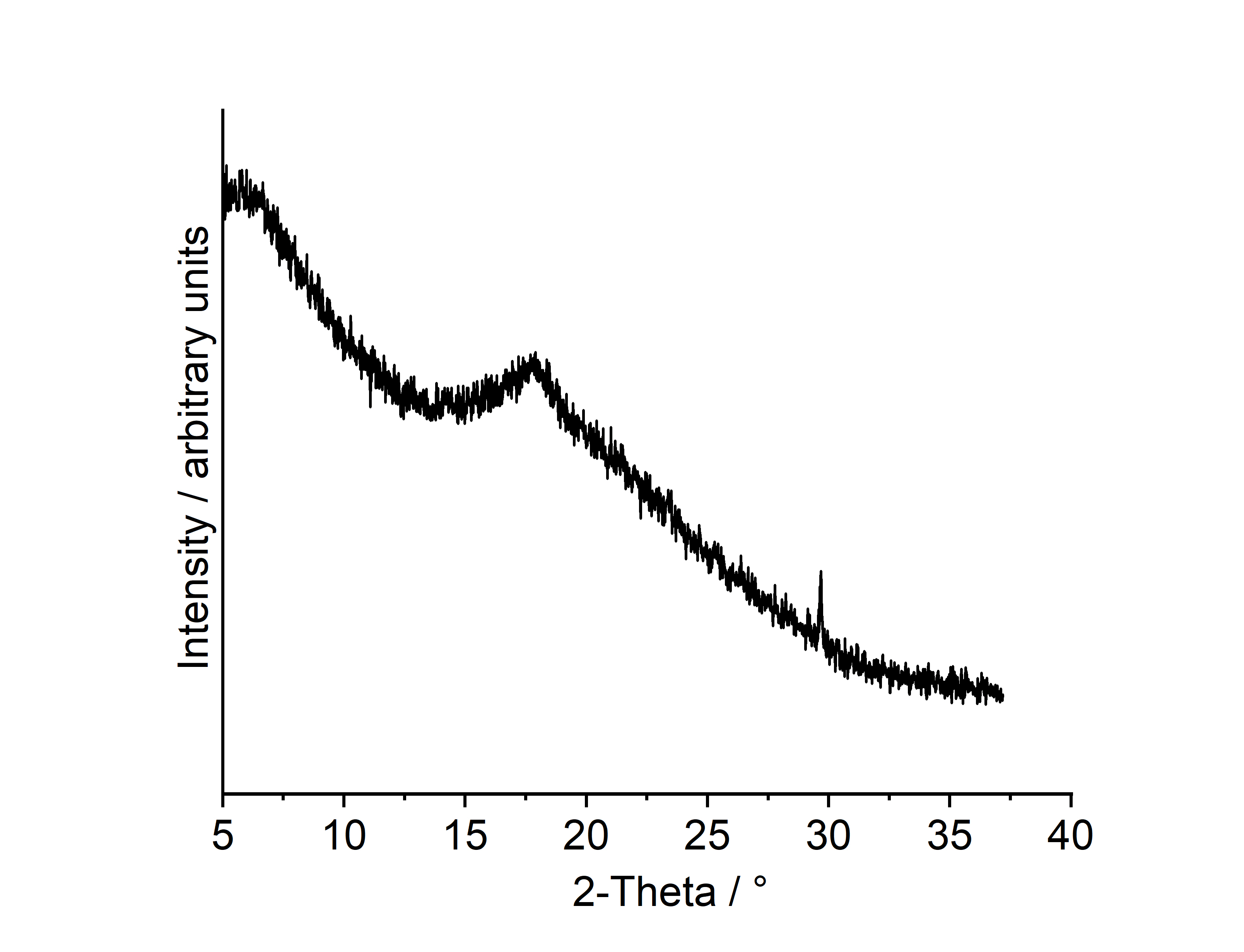

Supplement: Supplementary file 5 [file e-79-00216-sup5.jpg]
